# Benzimidazole-Derived B2 as a Fluorescent Probe for Bacterial Outer Membrane Vesicle (OMV) Labeling: Integrating DFT, Molecular Dynamics, Flow Cytometry, and Confocal Microscopy

**DOI:** 10.3390/ijms26104682

**Published:** 2025-05-14

**Authors:** Francisco Parra, Alexander Carreño, Evys Ancede-Gallardo, Diana Majluf, Jorge A. Soto, Romina V. Sepúlveda, Daniel Aguayo, María Carolina Otero, Iván L. Calderón, Fernando Gil, Juan A. Fuentes

**Affiliations:** 1 Laboratorio de Genética y Patogénesis Bacteriana, Centro de Investigación de Resiliencia a Pandemias, Facultad de Ciencias de la Vida, Universidad Andres Bello, Santiago 8370186, Chile; f.parralathrop@uandresbello.edu (F.P.); dmajlufosorio@gmail.com (D.M.); 2 Doctorado en Biotecnología, Facultad de Ciencias de la Vida, Universidad Andres Bello, Santiago 8370186, Chile; 3 Laboratory of Organometallic Synthesis, Departamento de Ciencias Químicas, Facultad de Ciencias Exactas, Universidad Andres Bello, Santiago 8370186, Chile; eancedeg@gmail.com; 4 Millennium Institute on Immunology and Immunotherapy, Laboratorio de Inmunología Traslacional, Centro de Investigación de Resiliencia a Pandemias, Facultad de Ciencias de la Vida, Universidad Andres Bello, Santiago 8370186, Chile; jorge.soto.r@unab.cl; 5 Center for Bioinformatics and Integrative Biology (CBIB), Facultad de Ciencias de la Vida, Universidad Andres Bello, Santiago 8370146, Chile; romina.sepulveda@unab.cl; 6 ANID—Millennium Nucleus in Data Science for Plant Resilience (PhytoLearning), Facultad de Ciencias de la Vida, Universidad Andres Bello, Santiago 8370146, Chile; 7 Instituto de Tecnología para la Innovación en Salud y Bienestar (ITISB), Facultad de Ingeniería, Universidad Andres Bello, Viña del Mar 2531015, Chile; daniel.aguayo@unab.cl; 8 Escuela de Química y Farmacia, Facultad de Medicina, Universidad Andres Bello, Santiago 7591538, Chile; maria.otero@unab.cl; 9 Laboratorio de RNAs Bacterianos, Centro de Investigación de Resiliencia a Pandemias, Facultad de Ciencias de la Vida, Universidad Andres Bello, Santiago 8370186, Chile; lcalderon@unab.cl; 10 School of Medicine, Faculty of Medicine, Universidad de los Andes, Santiago 7620001, Chile; frgil@uandes.cl; 11 Microbiota-Host Interactions & Clostridia Research Group, Center for Biomedical Research and Innovation (CIIB), Universidad de los Andes, Santiago 7620001, Chile

**Keywords:** outer membrane vesicles, OMVs, fluorescent labeling of OMVs, benzimidazole compound B2, confocal microscopy, flow cytometry, fluorescent dyes, intramolecular hydrogen bond

## Abstract

Bacterial outer membrane vesicles (OMVs) are nanoscale extracellular structures produced by Gram-negative bacteria that are critical for microbial biology and host-pathogen interactions and have great potential in biotechnological applications. Despite the availability of fluorescent dyes for OMV studies, many are repurposed from eukaryotic extracellular vesicle research and are not explicitly optimized for OMVs, leading to challenges in achieving consistent labeling, minimizing background noise, and preserving vesicle integrity during analyses. This study evaluates B2, a benzimidazole-derived fluorophore, for OMV labeling in advanced techniques like flow cytometry and confocal microscopy. OMVs were isolated from *Escherichia coli* strains BL21 and O157, and their integrity was confirmed using transmission electron microscopy (TEM). B2 staining protocols were optimized for OMVs, and fluorescence analyses revealed specific interactions with the vesicle membranes, reducing aggregation and enhancing signal uniformity. Flow cytometry indicated near-complete labeling efficiency (98–100%) with minimal background interference. Confocal microscopy further validated B2’s effectiveness, showing evident OMV internalization into epithelial HT-29 cells and compatibility with other fluorophores. Density functional theory (DFT) calculations, including Fukui function analysis, identified key electrophilic and nucleophilic regions in B2 that facilitate specific hydrogen bonding and polar interactions with membrane components. Non-covalent interaction (NCI) analysis revealed pronounced intramolecular hydrogen bonding along with discrete regions of weak van der Waals interactions. Molecular dynamics simulations suggest that B2 exhibits an affinity for both the hydrophobic core of the lipid bilayer and the core oligosaccharide region of the LPS layer, which collectively ensures sustained retention of the dye. The findings presented in this study position B2 as a valuable fluorophore for OMV research.

## 1. Introduction

Bacterial outer membrane vesicles (OMVs) are nanoscale, spherical structures released from the outer membrane of Gram-negative bacteria. These vesicles comprise a lipid bilayer encapsulating biomolecules such as proteins, lipids, and nucleic acids, mainly derived from the bacterial outer membrane and periplasmic space [1]. OMVs play critical roles in biological processes, including microbial communication, host–pathogen interactions, and immune modulation, contributing to maintaining gut microbiota balance [2,3,4]. Their ability to transport molecular cargo while protecting it from enzymatic degradation has made OMVs promising candidates for diverse biotechnological applications, including vaccine development and targeted drug delivery [5]. Recent studies have also uncovered OMVs with more complex architectures, such as outer–inner membrane vesicles containing cytoplasmic components, further expanding their functional repertoire [1,6].

Fluorescent labeling has become an essential tool for studying OMVs, enabling precise detection, quantification, and visualization in complex biological systems [7,8,9]. Techniques like flow cytometry and confocal microscopy have transformed OMV research by providing high-resolution insights into their functionality and interactions with host cells [7,8,9]. However, most fluorescent dyes commonly used in OMV studies have been adapted from extracellular vesicle (EV) research despite the distinct structural and biochemical properties of OMVs [10]. Unlike OMVs, EVs are released from eukaryotic cells and have distinct biochemical properties associated with their cellular origin [10]. While dyes such as PKH67, MemGlow, and DiO are widely employed for membrane labeling of vesicles, challenges persist, including non-specific binding, micelle formation, and signal variability [11,12,13,14]. Additionally, luminal dyes like calcein-AM, though reducing false positives, are less validated for OMVs, and genetic tags often alter vesicle properties, limiting their applicability [15]. Genetically encoded fluorescent and bioluminescent reporters, such as GFP, mCherry, and ThermoLuc, also serve as labeling options, though they come with limitations that may influence accuracy, including environmental pH sensitivity and the risk of altering vesicle properties during labeling [10,11,12,13,16]. Despite the widespread adoption of amphiphilic membrane dyes, several inherent drawbacks limit their utility for OMV studies. First, many of these probes self-assemble into vesicle-like micelles in aqueous buffer, producing artifactual fluorescence in the absence of true vesicles [17]. Second, labeling efficiencies are often very low (only ~5% of the vesicle population may become stained in some cases), undermining quantitative analyses [18]. Third, the broad size and cargo heterogeneity of OMV preparations lead to uneven dye uptake across subpopulations [19]. Fourth, endogenous autofluorescence from biological matrices can mask probe signals, creating a strong impetus for dyes with longer lifetimes and minimal background emission [20]. Finally, striking the correct balance between hydrophobic membrane affinity and aqueous solubility is challenging; misbalanced probes can precipitate or fail to integrate into lipid bilayers [21]. These challenges underscore the ongoing pursuit of novel fluorophores tailored for OMV applications, ensuring consistent and reproducible labeling in both microbiological research and clinical settings.

**Figure 1:**
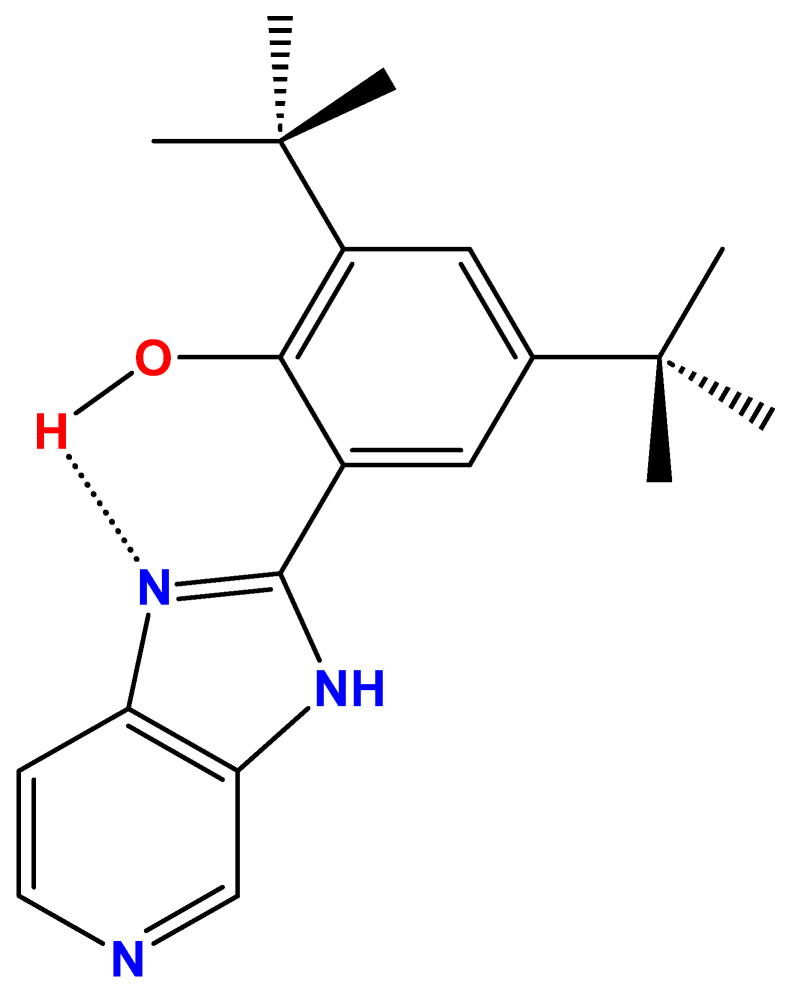
Chemical structure of B2 [4-di-*tert*-butyl-6-(3*H*-imidazo[4,5-*c*]pyridine-2-yl)phenol)]. The dotted line corresponds to an intramolecular hydrogen bond (IHB) [25,26,27].

Recent advances in small-molecule fluorophores, exemplified by the coumarin-based NO sensor ZPS-NO [22], the rhodamine–hydrazine NIR probe RBNE [23], and the coumarin–hemicyanine biothiol sensor BDP-CYS [24], underscore the power of rational small-molecule design. Extending this paradigm, we introduce B2, a benzimidazole-derived fluorophore (C_20_H_25_N_3_O), 2,4-di-*tert*-butyl-6-(3*H*-imidazo[4,5-*c*]pyridine-2-yl)phenol (Figure 1), which we evaluate as a novel fluorescent marker for bacterial outer membrane vesicles. B2 features a phenolic ring and an imidazo-pyridine group [25]. This structure includes an intramolecular hydrogen bond (IHB) between the hydrogen of the hydroxyl and a nitrogen atom of the imidazole ring, enhancing molecular stability, reducing vibronic relaxation, and improving fluorescent properties [25]. The steric hindrance that the *tert*-butyl groups provide significantly impacts the molecule’s conformation and electronic properties, enhancing its overall stability [25]. Furthermore, these *tert*-butyl groups create a hydrophobic region within the molecule, enabling it to exhibit both polar and non-polar characteristics. This duality facilitates potential intramolecular interactions, further influencing its structural and functional behavior [25]. B2 exhibits luminescent emission across various solvents at room temperature, with a large Stokes shift and suitable quantum yield, making it suitable for fluorescence staining in cellular environments and a promising candidate for biological imaging applications [25,26,27,28]. Given its effective staining properties for Gram-negative bacteria and cellular membranes, B2 demonstrates versatility in targeting a range of biological structures [25,26,27,28]. Based on these properties, we selected B2 to explore its potential as a fluorescent stain for OMVs, specifically assessing its application in flow cytometry and confocal microscopy.

In this work, we provide a comprehensive evaluation of B2 as an OMV probe. We first quantify its labeling efficiency and background suppression by flow cytometry, then visualize OMV uptake and localization in epithelial cells using confocal microscopy. Emission spectroscopy confirms B2’s stable fluorescence under repeated wash steps, and multi-dye spectral overlays demonstrate its seamless integration into multicolor imaging workflows. Finally, complementary computational studies, including density functional theory, non-covalent interaction analysis, and molecular dynamics simulation, reveal the molecular features underpinning B2’s high specificity and membrane affinity. Together, these data establish B2 as a robust, small-molecule marker for bacterial OMVs, offering a powerful new tool for both fundamental microbiological research and applied biomedical studies.

## 2. Results

### 2.1. B2 Interacts with Outer Membrane Vesicles (OMVs)

B2 demonstrates exceptional stability when dissolved in dimethyl sulfoxide (DMSO), establishing it as a practical and reliable fluorophore for various applications [25,26,27,28]. The compound retains its photophysical properties upon dissolution, including fluorescence intensity and emission profile, even after prolonged storage at low temperatures (e.g., −20 °C) [25,26]. Notably, this stability allows stock solutions of B2 to be prepared in advance, stored under appropriate conditions, and thawed with minimal loss of fluorescence performance or photochemical integrity [25,26]. Such resilience to freeze–thaw cycles highlights the robustness of B2, making it highly suitable for experiments that demand reproducible and consistent fluorescence. This characteristic positions B2 as an ideal candidate for long-term experimental workflows and multi-step imaging protocols.

B2’s interaction with OMVs was investigated using vesicles derived from two *Escherichia coli* strains: BL21, a model strain frequently employed in biotechnology [29], and O157, a pathogenic strain [30]. The selection of these strains allowed us to evaluate the versatility of B2 for staining OMVs in diverse biological contexts, with potential applications in both flow cytometry and confocal microscopy.

**Figure 2:**
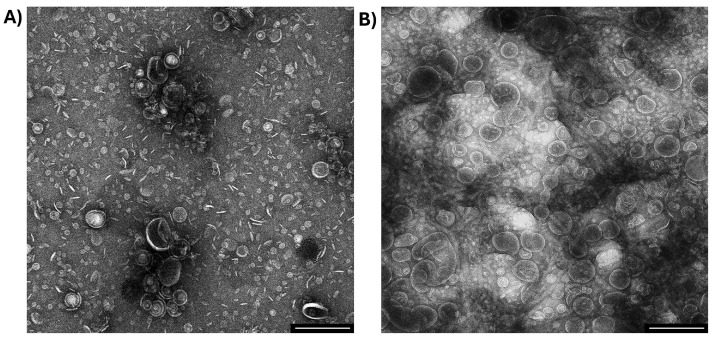
Transmission electron microscopy of OMVs produced by two *Escherichia coli* strains. *Escherichia coli* BL21 (**A**) or O157 (**B**) were cultured in LB to OD_600_ = 1.1 before extracting OMVs. The bar corresponds to 200 nm. A representative experiment is shown (*n* = 3).

Although OMV purification is a labor-intensive process requiring meticulous handling to preserve vesicle integrity and prevent contamination, our laboratory has extensive expertise in OMV extraction and purification [31,32,33]. This is evidenced by the high-quality transmission electron microscopy (TEM) images in Figure 2, which reveal spherical vesicles with well-defined bilayer membranes. These OMVs exhibit characteristic morphologies and size distributions consistent with the literature values, ranging from 20 to 200 nm in diameter [31,32,33]. The vesicles’ appearance underscores our purification protocol’s reliability, which ensures minimal contamination and structural preservation, which is critical for downstream fluorescence labeling and advanced microscopy applications.

**Figure 3:**
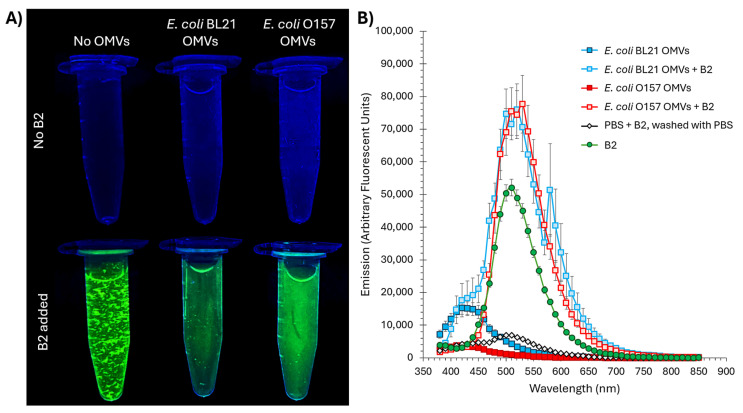
Interaction of B2 with *Escherichia coli* OMVs by fluorescence and aggregation assays. (**A**) UV-transillumination images illustrating aggregation and fluorescence of B2 in the presence or absence of OMVs. Top row: PBS alone, PBS + *E. coli* BL21 OMVs, and PBS + *E. coli* O157 OMVs (no B2) show no green fluorescence. Bottom row: B2 (1.0 mM) in PBS forms visible aggregates and patchy green emission (left); the addition of *E. coli* BL21 OMVs (center) or *E. coli* O157 OMVs (right) disperses these aggregates and produces uniform fluorescence, suggesting an interaction between B2 and OMVs. (**B**) Emission spectra (λ_ex_ = 350 nm) of: B2 alone (25 µM; positive control), PBS + B2 (0.1 mM, negative control processed identically to OMV samples to demonstrate wash efficiency), and OMVs (100 µg/mL protein) from BL21 or O157 stained with B2 (0.1 mM) and washed four times with PBS. Only the OMV samples treated with B2 or B2 alone exhibit a pronounced peak at ~500 nm, confirming specific retention of B2 by the vesicles. Data are mean ± SE (*n* = 3).

To evaluate B2 as a fluorescent marker for OMVs, we analyzed its behavior in both the presence and absence of OMVs derived from *E. coli* BL21 and O157 strains. Due to its partial hydrophobicity and limited solubility in aqueous solutions [25,26,27], B2 tends to form visible aggregates at high concentrations (more than 1.0 mM) when added to phosphate-buffered saline (PBS), as observed under UV transillumination (Figure 3A, bottom left). This aggregation is attributed to the hydrophobic surface area contributed by the *tert*-butyl groups on the phenolic ring, which reduces its affinity for water molecules [34]. Beyond their hydrophobic nature, *tert*-butyl groups are also characterized by their considerable bulk, creating significant steric effects. Their non-polarity arises from their composition of carbon and hydrogen atoms, which have similar electronegativities, resulting in a relatively uniform charge distribution [25,34]. Furthermore, their branched and symmetrical structure minimizes any significant dipole moment, reinforcing their apolar characteristics. Additionally, the phenolic hydroxyl group (–OH) in B2 forms intra- or intermolecular hydrogen bonds, promoting molecular clustering through stacking or aggregation. Such interactions could lead to stacking or clustering effects, promoting the aggregation of molecules as they interact more strongly with each other than with surrounding water molecules [35]. This aggregation is commonly observed with amphipathic or hydrophobic molecules in water and could be visually detected [35].

When OMVs from *E. coli* BL21 or O157 were introduced into the B2-PBS solution, a significant reduction in aggregation was observed, resulting in clearer solutions with uniform fluorescence (Figure 3A, bottom center and right). This behavior suggests that OMVs act as dispersing agents, interacting with B2 via hydrophobic or membrane-associated interactions. The vesicles likely provide a lipid bilayer interface stabilizing B2, reducing its tendency to aggregate and enhancing fluorescence uniformity. In control samples without B2 (Figure 3A, top row), no fluorescence was detected in PBS alone or PBS containing unstained OMVs, confirming that neither PBS nor OMVs contribute to the greenish fluorescence observed in the presence of B2. Importantly, the concentration employed for OMV staining in our experiments is 0.1 mM B2 (tenfold lower than the concentration used in the tube shown in Figure 3A, where precipitation is clearly visible), at which no visible precipitates form (see Figure S4), ensuring dye stability and homogeneity under assay conditions.

Fluorescence measurements further validated these interactions. Using emission scanning, we observed a prominent fluorescence peak at approximately 500 nm for OMV samples treated with B2 and washed four times with PBS to remove any residual B2 (Figure 3B). This emission aligns closely with the expected emission wavelength of B2 [25] (green in Figure 3B), confirming its retention and incorporation into the vesicles and showing that OMV association does not induce a measurable shift in emission. Control scans included unlabeled OMVs (no B2) processed through the same wash regimen, as well as PBS mixed with B2 and subjected to four washes as described in Materials and Methods; both controls exhibited only baseline fluorescence, demonstrating the efficacy of our washing procedure in removing unbound dye. The absence of significant signals in these controls underscores the specificity of B2’s interaction with OMVs.

These findings strongly suggest that B2 interacts with OMVs, likely through hydrophobic interactions or membrane association, resulting in a stable and evenly distributed fluorescence signal. The ability of B2 to retain fluorescence through subsequent washing steps further establishes its suitability as a fluorescent marker for OMV detection and analysis in diverse biological applications, including confocal microscopy and flow cytometry.

### 2.2. B2 Enables Reliable and Specific Detection of OMVs via Flow Cytometry

To evaluate the suitability of B2 as a fluorescent dye for detecting OMVs via flow cytometry, OMVs were extracted from *E. coli* strains BL21 and O157. The vesicles were stained with B2 and subjected to four washes with PBS to remove the unbound dye, ensuring specific fluorescence signals. Control samples included PBS alone, unstained OMVs, and PBS mixed with B2 (without OMVs) washed four times with PBS to assess background fluorescence and validate the washing protocol’s efficiency.

Flow cytometry was selected for this analysis because it provides high-throughput, quantitative evaluations of fluorescently labeled particles in complex biological mixtures. The reliable detection and characterization of OMVs are critical for advancing microbiological and clinical applications. As a fluorescent probe, B2 offers the potential to enhance the accuracy and specificity of vesicle quantification, addressing a crucial need in OMV-based research.

In flow cytometry, parameters such as SP (small particles) and SSC-A (side scatter-area) are essential for analyzing OMV complexity and granularity [36]. SSC-A distinguishes OMVs from other small particles or debris/noise in the sample, as light scattering correlates with vesicle complexity and structural integrity. Combined with SP settings, SSC-A optimizes the detection and resolution of submicron particles, ensuring accurate OMV characterization, even at their sub-micron size.

B2 fluorescence was detected using the laser and channel optimized for BV421, as B2’s excitation and emission spectra closely align with this fluorophore [37]. Both are efficiently excited by a 405 nm violet laser [25,26,37]. While B2 has a peak emission near 510 nm, its broad emission spectrum (450–600 nm) and strong fluorescence at 488 nm [25] make it compatible with the detection range of the flow cytometer (421–488 nm). The intrinsic compatibility of B2 with the cytometer’s optical setup allowed for efficient excitation and robust fluorescence detection without the need for additional laser configuration. These properties underscore B2’s versatility and suitability for flow cytometry applications.

**Figure 4:**
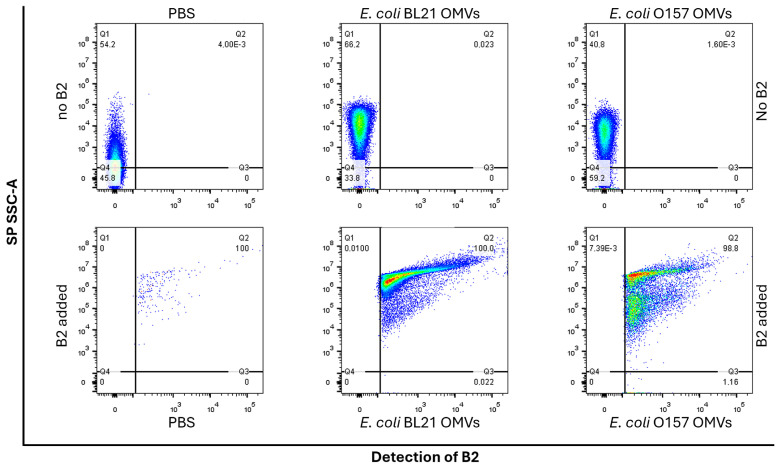
Flow cytometry analysis of OMVs labeled with B2. Flow cytometry was performed to analyze OMVs extracted from *E. coli* BL21 and O157 strains after labeling with B2. (**Top row**) Controls show low-complexity particles in PBS (**top left**) with no fluorescence, while unstained OMVs from BL21 (**top middle**) and O157 (**top right**) exhibit higher complexity due to their vesicular structure but lack fluorescence. (**Bottom row**) PBS mixed with B2 and washed to remove unbound B2 (**bottom left**) displays minimal fluorescence, establishing background noise likely due to B2 aggregation. B2-labeled BL21 OMVs (**bottom middle**) show 100.0% ± 0.0 fluorescence, while O157 OMVs (**bottom right**) display 98.8% ± 1.1 fluorescence with a slightly heterogeneous distribution. Analyses were performed using a FACSymphony™ A1 flow cytometer (BV421 channel) and FlowJo software v10.

Figure 4 demonstrates the results of flow cytometry analysis. The PBS-only control (Figure 4, top left) revealed low-complexity particles, likely background noise or minute impurities, with no detectable fluorescence. Similarly, unstained OMVs from both BL21 and O157 strains (Figure 4, top middle and right) showed higher complexity due to their vesicular structures but lacked fluorescence, confirming that OMVs themselves did not contribute to the fluorescent signal. PBS mixed with B2 washed four times with PBS (Figure 4, control, bottom left) exhibited minimal fluorescence, attributed to small aggregates of unbound B2. This finding validated the washing protocol’s efficiency and established the technique’s background fluorescence.

OMVs labeled with B2 showed strong fluorescence signals, confirming effective dye incorporation. BL21 OMVs (Figure 4, bottom middle) displayed nearly 100% labeling efficiency with a homogeneous fluorescence pattern, indicating uniform integration of B2 into vesicle membranes. In contrast, O157 OMVs (Figure 4, bottom right) exhibited slightly lower labeling efficiency (~98%) and a more heterogeneous fluorescence distribution, suggesting the presence of two distinct OMV subpopulations. These differences align with the known variability in OMV size and molecular composition across bacterial strains [38,39].

The results highlight B2’s specificity for OMVs, as evidenced by the clear separation between labeled and unlabeled samples and its ability to reveal vesicle heterogeneity. The combination of high labeling efficiency, low background noise, and compatibility with standard flow cytometry equipment positions B2 as a valuable tool for OMV research.

Finally, to evaluate whether protein contaminants such as flagella contribute to the B2 fluorescence signal, we compared vesicle preparations with and without flagella. Because the TEM of our *E. coli* OMVs (Figure 2) showed no visible flagella, we used *Salmonella enterica,* which produces abundant peritrichous flagella that often co-purify with OMV fractions [40], as a more stringent test. OMVs were isolated from the *Salmonella enterica* wild-type STH2370 strain and an isogenic Δ*fliC* mutant lacking flagellin. This approach enabled us to directly assess the impact of co-purifying flagellin on B2 staining. Both strains were cultured in LB to OD_600_ = 1.1, and OMVs were isolated using our standard ultracentrifugation protocol. Transmission electron microscopy (TEM) of the WT preparation revealed flagellar filaments co-purifying with OMVs (white arrowheads), whereas no filamentous structures were observed in the Δ*fliC* sample; in both cases, spherical vesicles were clearly visible (black arrowheads) (see Figure S5).

*Salmonella enterica* WT and Δ*fliC* OMVs were then stained with 0.1 mM B2 under identical conditions. Flow cytometric analysis showed nearly 100% fluorescence for both preparations, with no significant difference in labeling efficiency between the flagellated and non-flagellated samples (see Figure S5).

Because Δ*fliC* OMVs, entirely devoid of flagella, exhibit the same B2 signal as the WT vesicles, we conclude that B2 fluorescence arises from lipid-bilayer staining of OMVs rather than from co-purifying protein contaminants. This result not only supports the specificity of B2 for OMVs but also demonstrates its broad applicability across diverse Gram-negative species.

### 2.3. B2 Enables Precise Visualization of OMV Internalization in HT-29 Cells

OMVs from *Escherichia coli* have been extensively studied for their ability to internalize into epithelial cells, playing pivotal roles in host–pathogen interactions and maintaining intestinal homeostasis [41,42]. Confocal fluorescence microscopy remains a cornerstone technique for investigating these processes, leveraging fluorescently labeled OMVs to track their internalization and intracellular localization with high spatial resolution [43,44]. The identification of specific endocytic pathways has further illuminated the mechanisms of OMV entry, offering critical insights into how these vesicles interact with host cells [9,41,42]. To study these interactions, human colonic epithelial cell lines, such as HT-29, are commonly employed due to their ability to mimic the intestinal epithelium. These cell lines provide a robust and reproducible platform for investigating OMV internalization and its broader implications for gut health and disease [9,41,42].

**Figure 5:**
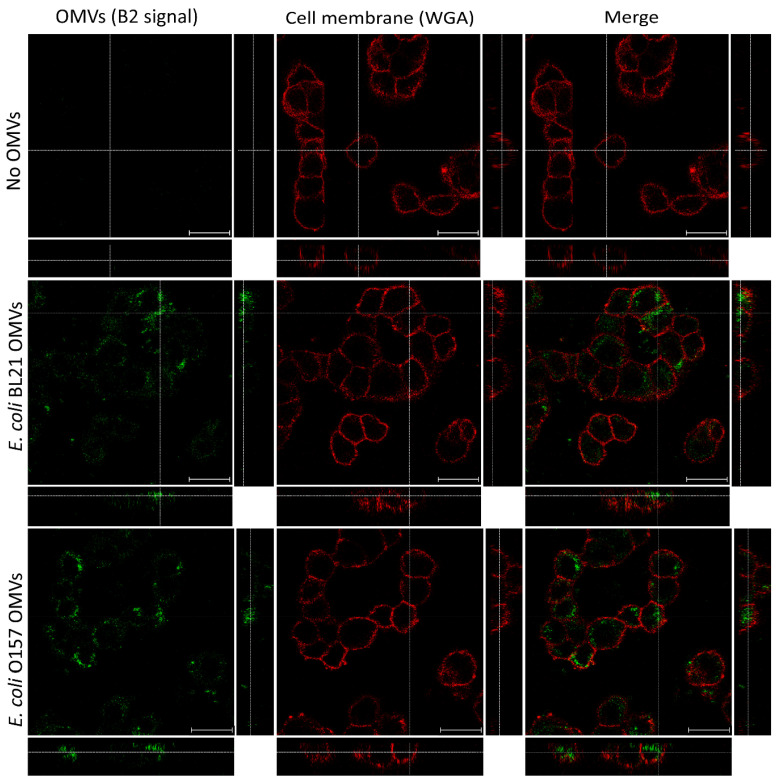
Confocal microscopy analysis of B2-labeled OMVs in HT-29 cells. The interaction and internalization of B2-labeled *E. coli* OMVs in HT-29 cells were visualized using confocal microscopy. (**Top row**) Control cells were treated with PBS mixed with B2, followed by four washes with PBS to remove the unbound dye, and no green fluorescence (B2 signal) was observed, confirming the specificity of the staining protocol. Cell membranes stained with wheat germ agglutinin (WGA, red) provide a structural reference, and the merged image shows no overlap between the red and green channels, validating the absence of non-specific fluorescence. (**Middle and bottom row**) Cells treated with *E. coli* BL21 and O157 OMVs, respectively, display strong green fluorescence (B2 signal, artificial color), with orthogonal projections predominantly confirming the intracellular localization (green signal beneath the red membrane in orthogonal views). Scale bars represent 20 µm.

In this study, we evaluated the potential of B2 as a fluorescent dye for visualizing OMVs during their interaction with HT-29 cells. Confocal fluorescence microscopy was employed to determine whether B2-labeled OMVs could be effectively detected and localized within these cells. Cell membranes were counterstained with wheat germ agglutinin (WGA) conjugated to Alexa Fluor^TM^ 647 (red), ensuring assay specificity and enabling precise localization of OMVs within cellular structures. Control experiments with PBS mixed with B2, followed by four washes with PBS to remove the unbound dye, revealed no non-specific fluorescence (Figure 5, top row). This result validated the specificity of the staining protocol, ensuring that any observed signal originated exclusively from OMV-specific interactions. The cell membranes, clearly delineated in red by WGA staining, served as a reliable structural reference. The merged image confirmed the absence of green fluorescence in the controls, further substantiating the absence of artifacts in the B2 channel.

On the other hand, strong green fluorescence was observed in HT-29 cells treated with OMVs derived from *E. coli* BL21 and O157 (Figure 5, middle and bottom rows), confirming the successful labeling of OMVs with B2. Orthogonal projections revealed intracellular localization, with green fluorescence signals detected beneath the WGA-defined red membrane. These findings demonstrate the uptake of OMVs by the epithelial cells and show intracellular trafficking pathways. The fluorescence distribution further supported OMV internalization, as confirmed by orthogonal projections and merged images that depicted their localization within the cells.

**Figure 6:**
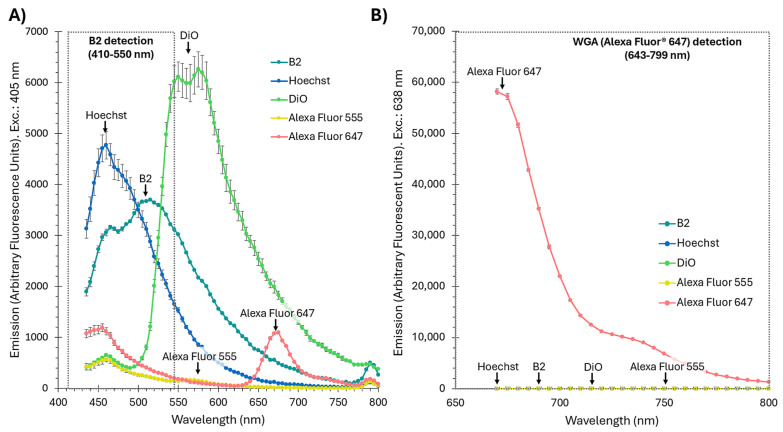
Emission spectra of fluorophores used for confocal microscopy to evaluate their compatibility. (**A**) Emission spectra of B2, Hoechst, DiO, Alexa Fluor^TM^ 555, and Alexa Fluor^TM^ 647 were recorded following excitation at 405 nm, the wavelength used for detecting B2 in confocal microscopy. (**B**) Emission spectra of the same fluorophores following excitation at 638 nm, the wavelength used for detecting WGA conjugated to Alexa Fluor^TM^ 647 in confocal microscopy. Alexa Fluor^TM^ 647 exhibited strong emission between 643 and 799 nm, with no detectable contribution from the other fluorophores, including B2. The gray boxes with dotted lines indicate the detection ranges used for B2 (410–550 nm) and Alexa Fluor^TM^ 647 (643–799 nm) during confocal microscopy. Error bars represent standard errors (*n* = 3).

To further evaluate the compatibility of B2 with other fluorophores used in confocal microscopy, the emission spectra of B2, Hoechst, DiO, Alexa Fluor^TM^ 555, and Alexa Fluor^TM^ 647 were recorded (Figure 6). DiO serves here as a representative of 488 nm-excited lipophilic probes (e.g., PKH67), which exhibit virtually identical spectral features. Under 405 nm excitation (Figure 6A), B2 exhibited a broad emission spectrum peaking at 510 nm, overlapping significantly with Hoechst and DiO within the 410–550 nm range. This overlap was experimentally confirmed, as the emission of B2 could not be distinguished from that of Hoechst or DiO under confocal microscopy or flow cytometry conditions, precluding their simultaneous use. In contrast, Alexa Fluor^TM^ 555 exhibited minimal emission under 405 nm excitation, while Alexa Fluor^TM^ 647 showed no detectable emission, demonstrating their compatibility with B2 for multi-fluorophore experiments (Figure 6A). When excited at 638 nm (Figure 6B), Alexa Fluor^TM^ 647 emitted strongly within the 643–799 nm range, with no significant contributions from B2 or other dyes, validating its use for membrane staining.

These results indicate that while B2 is incompatible with Hoechst and DiO (as well as with PKH67) due to spectral overlap, it is fully compatible with Alexa Fluor^TM^ 555 and Alexa Fluor^TM^ 647, enabling its use in multi-label experiments. The combination of B2 for OMV detection and Alexa Fluor^TM^ 647 for membrane staining offers a reliable, interference-free approach for confocal microscopy. Furthermore, the absence of fluorescence artifacts in controls and the strong, specific signals observed in OMV-treated cells underscore B2’s effectiveness as a fluorescent marker for confocal applications, enabling detailed studies of bacterial–host interactions.

### 2.4. Quantum Chemistry Analysis: Density Functional Theory (DFT) Fukui Function

Density functional theory (DFT) Fukui functions serve as robust descriptors for identifying the most reactive sites in a molecule for electrophilic and nucleophilic interactions [45,46]. In this study, calculating the Fukui functions for B2 is critical to elucidate its reactivity profile and to better understand its potential role as an effective fluorescent probe for OMV labeling. By precisely mapping the electrophilic (f^−^) and nucleophilic (f^+^) regions, we obtain a quantitative assessment of the electronic distribution across B2, with particular emphasis on the reactive centers associated with its benzimidazole and phenolic moieties.

**Figure 7:**
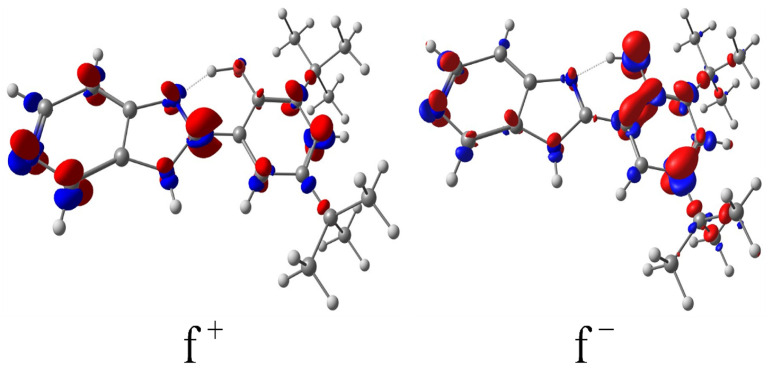
Nucleophilic and electrophilic Fukui functions of the frontier molecular orbital approximations for B2. The scalar fields represent the nucleophilic Fukui function f^+^ (r) and the electrophilic Fukui function f^−^ (r). These monophasic functions are positive and indicate sites of electrophilic and nucleophilic behavior, respectively. Red indicates areas with higher Fukui function values, which correspond to sites of greater reactivity.

According to molecular orbital theory, the frontier orbitals, namely the highest occupied molecular orbital (HOMO) and the lowest unoccupied molecular orbital (LUMO), determine the electrophilic and nucleophilic behavior of atomic centers. Specifically, the nucleophilic character is largely governed by the electron density in the HOMO, while the electrophilic character is influenced by the LUMO electron density [47]. The derived Fukui functions, which are inherently positive, represent the electron density contributions from the HOMO (for f^−^) and LUMO (for f^+^). As shown in Figure 7, the electrophilic Fukui map (f^−^) shows that the most reactive sites appear on heteroatoms and on the phenolic ring’s *ortho* and *para* positions, reflecting the activating effect of the phenol substituent. This aligns with well-established chemical principles, indicating that phenolic groups direct incoming electrophiles to these positions [48,49]. In contrast, the nucleophilic Fukui function (f^+^) highlights increased reactivity on the carbons adjacent to the nitrogen atoms in the imidazole-pyridine segment, correlating with the higher positive charge density in that region.

From a broader perspective, these findings support the notion that B2’s structure incorporates well-defined reactive centers, which help explain its stable interactions with bacterial outer membrane vesicles. The highlighted nucleophilic and electrophilic sites provide a rationale for the favorable interplay of B2 and OMVs, where both hydrophobic and potential hydrogen-bonding interactions may occur. In this sense, the Fukui function data support the conclusion that B2’s electronic configuration underpins its effectiveness as a fluorescent dye for OMV labeling without introducing additional reactivity that could compromise vesicle integrity or labeling fidelity.

### 2.5. Quantum Chemistry Analysis: Non-Covalent Interaction Index (NCI)

A comprehensive understanding of non-covalent interactions in fluorescent probes is relevant for better understanding their stability, electronic properties, and overall suitability for biological applications [50,51]. In this study, we employed the non-covalent interaction (NCI) index to dissect the key forces governing the conformation and intramolecular bonding of B2.

**Figure 8:**
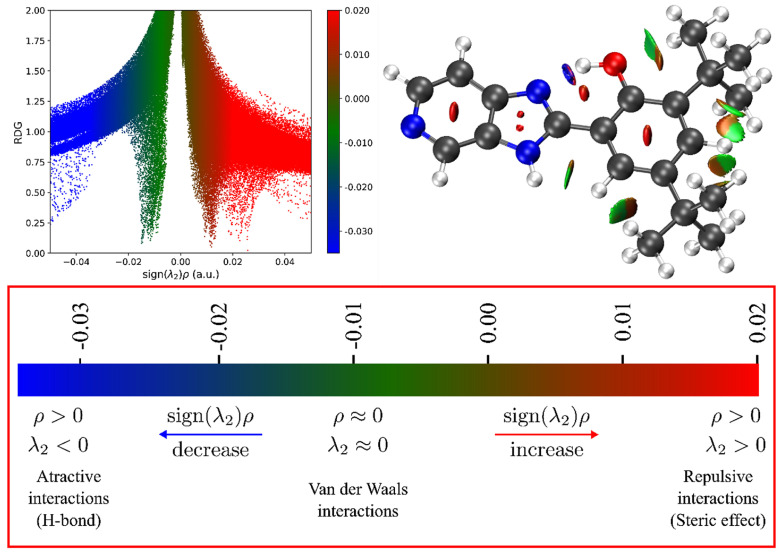
Non-covalent interaction (NCI) analysis of B2 (**top**). **Bottom**: the NCI plot gradient isosurface (0.6 a.u.) for five local and global minima structures (kcal/mol). The NCI color scale is −0.02 < λH > 0.02 a.u. These calculations were performed at the reduced density gradient (RDG) level. NCI analysis was conducted using Multiwfn 3.8, and isosurface visualization was performed in VMD 1.9.4a57. The RDG vs. sign(λ_2_)ρ plot was generated in Python version 3.12 using matplotlib.

The NCI index leverages electron-density data and its derivatives to locate and classify non-covalent interactions (NCIs) purely on the basis of electron density [52,53]. Specifically, the reduced density gradient is analyzed in low-density regions, while each point is colored according to the sign of the second eigenvalue of the electron density Hessian (λ). This scheme enables rapid differentiation between attractive forces (λ < 0, typically hydrogen bonds), weak or van der Waals interactions (λ ≈ 0), and repulsive interactions (λ > 0). Figure 8 (bottom panel) illustrates the NCI plot gradient isosurface (0.6 a.u.) for five optimized local and global minima structures of B2, color-coded to represent the type and strength of the interactions (blue to red). These calculations reveal a strong intramolecular hydrogen bond (IHB) between the phenolic –OH and the nitrogen in the imidazole ring, evidenced by a pronounced negative λ value (λH < 0) in the corresponding region. This attractive interaction confers structural rigidity on B2 by minimizing conformational freedom and vibrational relaxation, which in turn enhances its fluorescence quantum yield. The presence of the IHB is consistent with complementary spectroscopic data, including a downfield ^1^H NMR signal at ~13.6 ppm.

Beyond the IHB, the *tert*-butyl substituents give rise to both weak van der Waals contacts (green areas) and localized steric repulsions (red regions) (Figure 8). Notably, these steric effects can protect the fluorescent core from quenching agents and reduce undesirable intermolecular interactions in solution. Additionally, repulsive interactions appear in the central rings, aligning with the rigid nature of the aromatic system. Altogether, the balance of strong intramolecular hydrogen bonding, localized van der Waals contacts, and steric hindrance underpins the high photostability and low susceptibility to photobleaching observed for B2 [26].

The intramolecular non-covalent interactions revealed by NCI analysis are central to explaining B2’s performance as an OMV label. The stable conformation and reduced non-radiative decay pathways imparted by the IHB support its bright emission and large Stokes shift, both of which are critical for flow cytometry and confocal microscopy. Moreover, the coexistence of polar (IHB region) and non-polar (*tert*-butyl region) sites in the same molecule likely promotes favorable partitioning into OMV membranes, mitigating the tendency of B2 to aggregate in purely aqueous environments. Thus, the NCI analysis not only confirms the strong intramolecular interactions in B2 but also correlates directly with its robust fluorescence properties and high specificity for OMV labeling, aligning well with the overarching objectives of this study.

### 2.6. B2–OMV Interactions Characterized Through Molecular Dynamics Simulations

In order to complement the experimental findings described in previous sections, molecular dynamics simulations were performed to elucidate the molecular basis for B2’s retention in bacterial OMVs. Our simulation model mimicked a Gram-negative outer membrane featuring an outer leaflet composed of lipopolysaccharides (LPS) and an inner leaflet containing a mixture of phospholipids and cardiolipins. In each of the three simulation replicas, six B2 molecules were randomly positioned in the solvent adjacent to the membrane, allowing us to assess their spatial distribution and interaction dynamics over time.

**Figure 9:**
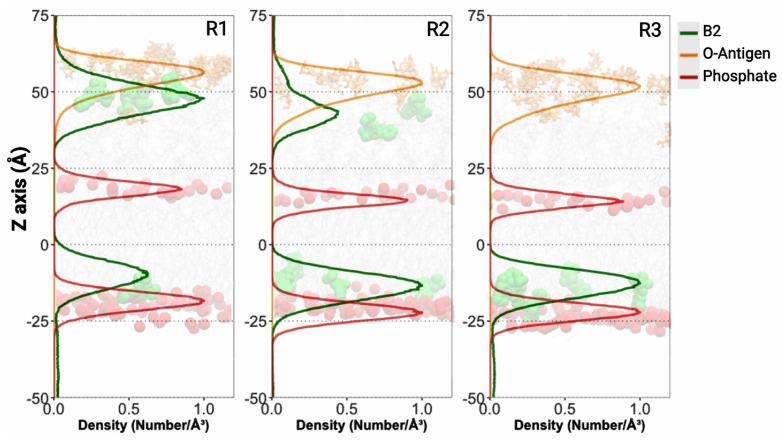
Density profile along the *Z*-axis for the simulated system. The plot shows the normalized number density (number/Å^3^) as a function of the Z-coordinate, averaged over the trajectory. Different colored lines represent distinct molecular species or structural components within the system: B2 (green), O-antigen (orange), and bilayer phosphate groups (red). The observed density variations reflect the structural organization of the system and potential molecular interactions at the membrane interface.

All B2 molecules successfully engaged with the OMV model during the simulations. In several replicas, a subset of B2 molecules (observed as 2/6, 4/6, and 6/6 per replica) penetrated the membrane’s hydrophobic core. This suggests that B2 possesses an intrinsic affinity for the hydrophobic regions, likely due to its partially non-polar character. Such interactions would contribute to the fluorophore’s stable retention within the OMV membrane, as also evidenced by the robust labeling observed in our flow cytometry and confocal microscopy experiments. Interestingly, the remaining B2 molecules were found to preferentially accumulate along the outer core of the LPS. Specifically, in different replicas, B2 localization near Heptose IV residues was noted (with patterns of 4/6, 2/6, and 0/6 across replicas) (Figure 9, Videos S1–S3). This distribution pattern implies that interactions, potentially via hydrogen bonding between B2’s hydroxyl groups and the sugar moieties of the LPS, play a significant role in its retention. Moreover, the observed accumulation may indicate a tendency toward aggregate formation in this region, which could impede or make further diffusion of B2 molecules difficult due to entropic barriers imposed by the complex LPS architecture. This phenomenon might explain why some B2 molecules remain in the outer regions of the membrane rather than fully penetrating the hydrophobic core.

Collectively, these simulations provide a mechanistic framework that reinforces our experimental observations. The dual interaction of B2 with both the hydrophobic core and the LPS sugars, along with the potential for aggregate formation, may contribute to its stability as an OMV marker. Furthermore, these observations deepen our understanding of B2’s partitioning behavior and interactions within membrane environments, which could have implications for the design and optimization of OMV-based technologies.

## 3. Discussion

Outer membrane vesicles (OMVs) are integral to bacterial physiology and host–pathogen interactions. Their capacity to mediate intercellular communication, deliver biomolecules, and modulate host responses has positioned them as a focal point in microbiological and biomedical research [2,4,54]. However, the nanoscale size (20–200 nm) and complex bilayer structure of OMVs present challenges for their detection and visualization, necessitating advanced tools such as reliable fluorescent dyes tailored for these applications [12,55,56,57,58].

In this study, we evaluated the benzimidazole-derived compound B2 [25,26,27] as a fluorescent marker for OMVs, focusing on its applicability in flow cytometry and confocal microscopy, two key techniques for OMV quantification and visualization [9,59,60,61]. These methodologies demand fluorophores with high specificity, low background interference, and compatibility with multicolor systems [18]. By addressing these requirements, we aimed to position B2 as a practical and effective tool for advancing OMV research with implications for fundamental science and therapeutic development.

The chemical structure of B2 (Figure 1) underpins its unique fluorescence properties. The intramolecular hydrogen bond (IHB) between the phenolic hydroxyl group and the nitrogen atom in the imidazopyridine moiety enhances structural rigidity, reducing vibrational relaxation and non-radiative decay, which improves its fluorescence quantum yield and spectral stability [25,26,27]. Additionally, the *tert*-butyl groups shield the phenolic ring from quenching agents, contributing to B2’s remarkable resistance to photobleaching and blinking [25,26,27]. These properties, coupled with a large Stokes shift and good quantum yield, make B2 ideal for fluorescence detection, as they reduce spectral overlap and improve signal-to-noise ratios, particularly in high-resolution imaging [62,63,64].

The biocompatibility of B2 further highlights its potential as a fluorophore. Experimental evidence shows that B2 is non-toxic to cell lines such as HEK-293, SKOV-3, and HeLa, as well as to bacteria like *Salmonella enterica* [25,26]. Moreover, B2 has proven effective for staining *Pseudomonas aeruginosa* biofilms and intracellular organelles such as the endoplasmic reticulum and Golgi apparatus in HeLa cells, with internalization occurring without the need for permeabilization [25,26,28]. Additionally, B2 has been employed as an ancillary ligand in the complex *fac*-[Re(CO)_3_(deeb)B2]^+^ for protein staining, further demonstrating its versatility across a wide range of biological applications [27]. On the other hand, the photostability of B2 was previously demonstrated in HeLa cells (50 µg/mL; 60 min continuous illumination) without measurable photobleaching or blinking [26]. This remarkable resistance arises from its IHB-stabilized scaffold and *tert*-butyl steric protection, ensuring bright, stable fluorescence under prolonged excitation [26]. These findings position B2 as a robust and adaptable fluorescent marker suitable for imaging diverse biological systems, from bacterial cells to eukaryotic organelles.

The transmission electron microscopy (TEM) images in Figure 2 confirm the successful isolation and structural integrity of OMVs extracted from *E. coli* BL21 and O157. TEM is a critical tool for characterizing OMVs, providing insights into size, morphology, and membrane composition [31,65]. These well-defined vesicles with distinct bilayer membranes were of suitable quality for downstream fluorescence-based detection and imaging experiments, ensuring the reliability of subsequent analyses.

The interaction between B2 and OMVs, as demonstrated in Figure 3, highlights its utility as a fluorescent marker. The disappearance of B2 aggregates in the presence of OMVs indicates that the vesicles act as dispersing agents, likely through hydrophobic interactions with B2’s non-polar regions. This interaction stabilizes the fluorophore, reducing aggregation and enhancing fluorescence uniformity, which are critical attributes for fluorescence-based applications. These findings underscore the amphipathic nature of OMVs, whose lipid bilayers provide an ideal interface for incorporating partially hydrophobic fluorophores like B2 [5,66,67].

Fluorophore aggregation in aqueous environments is often associated with fluorescence loss, a phenomenon known as aggregation-caused quenching (ACQ) [68,69]. This is typically driven by restricted photoinduced electron transfer, non-radiative decay pathways, or conformational changes in the aggregated state, which disrupt or diminish the molecule’s ability to emit light effectively [68,69]. Despite its aggregation tendency in aqueous environments, B2 exhibits a notable resistance to aggregation-caused quenching (ACQ) [25]. This property is attributed to its structural design, including the steric hindrance provided by *tert*-butyl groups, which reduces π-π stacking interactions and minimizes fluorescence loss [25]. While aggregation in water remains a limitation, the interaction with OMVs effectively mitigates this issue, resulting in stable and uniform fluorescence signals (Figure 4 and Figure 5).

Nevertheless, aggregation still occurs due to the compound’s partial hydrophobic nature, driven by limited solubility in polar solvents. In this context, further optimization of its structure could unlock greater potential for its use in complex biological systems. Chemical modification presents an effective avenue to reduce aggregation and enhance the photophysical properties of B2. For example, incorporating hydrophilic functional groups, such as carboxylic acids, sulfonic acids, or phosphonic acids, could improve its water solubility and minimize the formation of aggregates [70,71]. Alternatively, non-ionic substituents, such as poly(ethylene glycol) (PEG) chains, could increase hydrophilicity while maintaining the integrity of B2’s fluorescence [70,71].

The flow cytometry results (Figure 4) demonstrated nearly complete labeling efficiency for OMVs derived from *E. coli* BL21 (100%) and O157 (~98%). This robust interaction between B2 and OMVs is likely mediated by hydrophobic interactions and potential hydrogen bonding with phospholipid head groups. Control experiments further confirmed this specificity: in untreated PBS, the cytometer detected low-complexity scatter events (buffer particulates and electronic noise) without any fluorescence; PBS containing OMVs showed increased side-scatter signals consistent with vesicles but still no fluorescence; and the PBS + B2 control, processed through the identical staining and four-wash regimen, yielded only rare, high-intensity fluorescent aggregates that triggered threshold adjustments and eliminated background scatter, resulting in a lower overall event count. Thus, minimal background fluorescence in the washed PBS + B2 control validates the washing protocol and gating strategy, ensuring that all measured fluorescence arises exclusively from OMV-bound B2. As observed in the TEM images (Figure 2), vesicles from *E. coli* strains exhibited distinct apparent size distributions, a heterogeneity mirrored by flow cytometry (Figure 4). Taken together, these data suggest that B2 staining preserves OMV integrity and natural heterogeneity and can be leveraged in future studies to dissect vesicle subpopulations. These findings underscore the specificity and reliability of B2 for OMV characterization in flow cytometry.

Although numerous studies have investigated the challenges of using flow cytometry and fluorophores for analyzing eukaryotic extracellular vesicles (EVs) [72,73,74,75,76], significant hurdles persist. Key issues include non-specific labeling, which can result in false positives; the aggregation of lipophilic dyes into micelles, complicating data interpretation; and signal variability caused by inconsistent dye incorporation. Additionally, the size detection limits of many cytometers hinder the accurate analysis of smaller vesicles (<200 nm), further restricting EV research [72,73,74,75,76]. In contrast, the fluorescent labeling and characterization of OMVs via flow cytometry remain relatively underexplored. Limited research has been devoted to developing and evaluating fluorophores specifically optimized for OMVs despite their distinct structural and compositional features compared to EVs. This gap underscores the need for tailored approaches, such as the use of B2, to advance OMV research and its applications in microbiology and host–pathogen interaction studies. This need is particularly pressing given the growing body of research employing flow cytometry to analyze OMV interactions with mammalian cells, immune responses, and drug delivery systems [77,78,79,80].

Confocal microscopy results (Figure 5) further validated the utility of B2 for labeling OMVs. The orthogonal projections confirmed intracellular localization, with clear green fluorescence signals from B2-labeled OMVs beneath the WGA-defined red membrane. This localization demonstrates OMV uptake by epithelial cells and suggests intracellular trafficking, showing a similar pattern to previously reported studies using other fluorophores [81]. The absence of background fluorescence in the controls underscores the specificity of B2, as the unbound dye was effectively removed during the washing steps. These findings align with previous studies showing the internalization of OMVs via endocytic pathways [41,42].

Figure 6 highlights the photophysical robustness of B2, particularly its compatibility with Alexa Fluor^TM^ 555 and Alexa Fluor^TM^ 647 for multi-fluorophore experiments. While B2 exhibited a broad emission spectrum that overlapped significantly with Hoechst and DiO, it demonstrated negligible spectral overlap with Alexa Fluor^TM^ 555 and Alexa Fluor^TM^ 647, enabling their simultaneous use in confocal microscopy. The absence of spectral interference ensures clean and independent detection of fluorescence signals, validating B2’s suitability for dual-label experiments.

Although spectral overlap limits the simultaneous use of B2 with Hoechst and DiO, future strategies could mitigate these challenges. Narrowband filters or spectral unmixing software could enhance the separation of overlapping signals in high-resolution imaging [82].

Quantum chemistry analyses provide valuable insights into B2’s effectiveness as an OMV labeling agent. Electrophilic regions, primarily located at the *ortho* and *para* positions of the phenolic ring, are complemented by nucleophilic areas near the imidazo-pyridine moiety. This distinct spatial separation of reactivity suggests that B2 interacts specifically with OMV membranes through a combination of hydrogen bonding and hydrophobic contacts, ensuring high labeling fidelity without compromising vesicle integrity. Moreover, benzimidazole compounds, owing to their structural similarity to nucleotides, readily interact with typically anionic bacterial membranes. The inherent electrophilic and nucleophilic regions in the benzimidazole structure facilitate effective binding and disruption of the membrane [83,84].

The NCI analysis reveals a pronounced negative λ value between the phenolic –OH and the imidazopyridine nitrogen, confirming a strong intramolecular hydrogen bond. This hydrogen bonding rigidifies the molecular structure, effectively reducing non-radiative decay and enhancing fluorescence, a crucial factor for reliable imaging in flow cytometry and confocal microscopy. Furthermore, the bulky *tert*-butyl groups in B2 generate weak van der Waals interactions along with localized steric repulsions that protect the fluorescent core. This steric shielding helps to reduce aggregation in aqueous environments by preventing the close packing of molecules, a prerequisite for strong intermolecular attractions that can lead to aggregation. Similar effects have been observed in related benzimidazole derivatives, where the presence of *tert*-butyl groups results in significant dihedral angles between aromatic rings and modulates rather than completely prevents aggregation [85,86].

Our molecular dynamics simulations show that B2 exhibited an affinity for both the hydrophobic core of the lipid bilayer and the core oligosaccharide region of the LPS layer, highlighting its potential to interact with multiple OMV membrane components. Additionally, and in line with previous studies on eukaryotic membranes, benzimidazole derivatives have been shown to form hydrogen bonds with the polar head groups of phospholipids, effectively anchoring the compound at the membrane interface and enhancing its stability within the bilayer [87]. Such interactions likely prevent the rapid dissociation of the dye, ensuring the retention of fluorescence over time.

Notably, our findings indicate that B2 preferentially localizes near specific sugar residues, particularly the Heptose IV moieties, suggesting that hydrogen bonding or stacking interactions with LPS sugars play a critical role in its retention (Videos S1–S3). To our knowledge, no published studies have previously described the interaction between benzimidazole compounds and LPS. This gap is significant because our data suggest that B2 not only interacts with the hydrophobic core but also exhibits specific interactions with the LPS outer core through polar contacts with sugar residues. This novel binding mechanism likely contributes to its stable incorporation into bacterial outer membranes.

This dual affinity broadens the retention mechanism and may reduce the diffusion of B2 molecules into deeper membrane regions, thereby preserving the fluorescent signal localized in the vesicle’s boundary membrane. The complex architecture of the LPS outer core, coupled with its relatively hydrophobic environment compared to the solvent, likely facilitates localized B2 aggregate formation through hydrophobic interactions. Such aggregates might further limit dye mobility by creating entropic barriers, which are beneficial in OMV labeling by contributing to a stable and persistent fluorescent signal.

Although a formal Kd was not determined here due to the heterogeneous and multivalent nature of OMV surfaces, the near-quantitative labeling and stringent wash resistance of B2 suggest a high-affinity interaction. Future work will focus on fluorescence–titration experiments using well-defined LPS or synthetic lipid-bilayer vesicles to derive precise binding constants and further elucidate the thermodynamics of B2-membrane interactions. Furthermore, while B2’s partitioning into lipopolysaccharide-rich Gram-negative outer membranes has been shown, its affinity for other lipid-rich envelopes (e.g., Gram-positive cell walls) remains to be explored. Such studies lie beyond our present scope but represent a logical next step to define the full spectrum of B2’s bioimaging applications.

## 4. Materials and Methods

### 4.1. Synthesis and Characterization of B2

B2 (C_20_H_25_N_3_O, MW: 323.43 g/mol), identified as 2,4-di-*tert*-butyl-6-(3H-imidazo[4,5-c]pyridine-2-yl)phenol (Figure 1), was synthesized via a condensation reaction between phenyl-3,5-di-*tert*-butyl-2-hydroxybenzoate and 3,4-diaminopyridine, following previously established protocols [25,26,27,88]. Starting materials, sourced commercially, were used without further purification. The reaction used nitrobenzene (Sigma-Aldrich, St. Louis, MO, USA) as the solvent under controlled conditions, facilitating condensation. This synthesis was adapted from the method developed by Benisvy et al. for related benzimidazole-based compounds [88,89]. The starting materials, phenyl-3,5-di-*tert*-butyl-2-hydroxybenzoate, and 3,4-diaminopyridine, were used in a 1:1 molar ratio with nitrobenzene as the solvent. The mixture was refluxed with vigorous agitation for over 4 h under an inert atmosphere. The product was then chromatographed using an organic solvent with increasing polarity. The polar fraction was evaporated to yield a brownish-yellow solid. Finally, the product was recrystallized from ethanol, resulting in a yield of less than 30%.

The ^1^H NMR and HHCOSY assays were recorded on a Bruker AVANCE 400 spectrometer at 400 MHz and 25 °C in deuterated DMSO with TMS. These techniques were employed to confirm the structure of B2. The ^1^H NMR, HHCOSY spectra, and expanded aromatic zone of B2 displayed the expected values (Figures S1–S3). The most significant frequency bands in the FT-IR spectrum appear at 3440 cm^−1^ and 1627 cm^−1^, corresponding to the –OH group forming an intramolecular hydrogen bond (IHB) and the –C=N– group of the imidazole, respectively. ^1^H NMR (400 MHz, DMSO-d6): δ= 13.60 [s; O-H], 8.98 [s], 8.35 [d; J = 5.3 Hz], 8.00 [s], 7.68 [d; J = 4.7 Hz], 7.38 [d, J = 1.9 Hz], 1.43 [s; 9H; *tert*-butyl]; 1.33 [s; 9H; *tert*-butyl]. In the ^1^H NMR spectrum, a low-intensity narrow signal at approximately 13.6 ppm is attributed to the IHB (Figure S3). Other aromatic protons appeared between where the aromatic pyridine moiety protons appear at 8.94 ppm as a singlet and two doublets at 8.36 ppm and 7.61 ppm. The phenolic ring moiety protons were assigned at 7.98 ppm and 7.48 ppm, respectively. The *tert*-butyl protons appear at 1.48 ppm and 1.36 ppm due to a loss of symmetry of the molecules.

Upon completion, the product was purified and characterized extensively. The melting point of B2 was recorded at 311–312 °C, consistent with reported values [25]. A whole structural characterization employed a combination of techniques, including ^1^H and ^13^C-NMR spectroscopy, HHCOSY, FT-IR spectroscopy, and electron impact mass spectrometry (EI-MS) to confirm the molecular composition and bonding patterns [25,26].

B2 demonstrated notable luminescent properties, emitting efficiently at room temperature across various solvents. In acetonitrile (Sigma-Aldrich, St. Louis, MO, USA), the compound exhibited an excitation maximum at 335 nm and an emission peak around 510 nm, with a large Stokes shift [25,26,27,28]. Its quantum yield was measured at 0.21, indicating efficient light emission upon excitation consistent with expected values [25,26,27,28].

### 4.2. Bacterial Strains, Media, and Culture Conditions

*Escherichia coli* BL21 and *Escherichia coli* O157 strains were utilized as outer membrane vesicle (OMV) sources. Cultures were routinely grown in Luria-Bertani (LB) medium, composed of Bacto tryptone (10 g/L, Gibco, Thermo Fisher, Waltham, MA, USA), Bacto yeast extract (5 g/L, Gibco, Grand Island, NY, USA), and NaCl (5 g/L, Winkler, Lampa, Chile) in distilled water, and incubated at 37 °C with shaking. Agar (15 g/L, Thermo Fisher, Waltham, MA, USA) was added to the medium when necessary.

### 4.3. OMV Isolation and Quantification

OMVs were isolated as previously described with few modifications [90]. Briefly, strains were grown in 1 L of LB medium with shaking (37 °C, 160 rpm) to OD = 1.1–1.3 before being centrifuged for 10 min at 5400× *g* at 4 °C. The resulting pellet, containing intact cells and large debris, was discarded to remove parent bacteria. The supernatant was then passed through a 0.45 µm filter (0.45 μm, Nalgene^TM^, Thermo Fisher, Waltham, MA, USA) to eliminate any remaining cells or aggregates larger than 450 nm, while allowing sub-micron OMVs (20–200 nm) to pass. This filtrate was concentrated and diafiltered using a tangential-flow cassette with a 100 kDa MWCO (nominal pore size ~10 nm, Sartorius, Göttingen, Germany), which retains OMVs and removes soluble proteins and small molecules. The retentate was subsequently ultracentrifuged (Sorvall™ WX, Rotor AH-629, Thermo Fisher, Waltham, MA, USA) at 150,000× *g* for 3 h at 4 °C. The supernatant was discarded, and the pellet was gently resuspended in 1 mL PBS (phosphate-buffered saline, Gibco, Thermo Fisher, Waltham, MA, USA). Aliquots of OMVs were stored at −80 °C until use. OMV protein content was quantified by BCA assay as described [32].

### 4.4. Transmission Electron Microscopy (TEM) of OMVs

OMVs were observed by TEM, as previously reported [31,32,33]. Briefly, OMV extracts were bound to formvar-coated slot grids (EMS, Hatfield, PA, USA), stained with 1% aqueous uranyl acetate for 1 min (EMS, Hatfield, PA, USA), and viewed with a Talos F200C transmission electron microscope (Thermo Fisher, Waltham, MA, USA).

### 4.5. OMV Fluorescent Labeling with B2

OMVs were stained with the dye B2 following a protocol adapted from the lipophilic dye DiO [14,91]. Specifically, 500 µg of OMVs, quantified by protein content, were resuspended in phosphate-buffered saline PBS (phosphate-buffered saline, Gibco, Thermo Fisher, Waltham, MA, USA), gauged at 990 µL and combined with 10 µL of a B2 10 mM solution (1% *v*/*v*, B2 final concentration of 0.1 mM). The OMV-B2 mixture was incubated in the dark at 37 °C for 1 h to facilitate membrane integration of the dye. Post-incubation, unbound B2 was removed by washing the stained OMVs four times with 4 mL of PBS (phosphate-buffered saline, Gibco, Thermo Fisher, Waltham, MA, USA), utilizing a 4 mL 100 kDa molecular weight cutoff (MWCO) filtration column (Amicon^TM^, Merck, Darmstadt, Germany). Subsequently, the stained OMVs were resuspended in fresh PBS and stored at 4 °C with Halt^TM^ protease inhibitor cocktail 1× (Thermo Fisher, Waltham, MA, USA) for downstream applications. As a control, the staining protocol was replicated using PBS alone instead of OMVs, applying the same incubation, staining, and washing steps. This control procedure ensured that any residual B2 was effectively removed, validating the washing protocol’s efficacy in eliminating unbound dye.

The B2 staining protocol is optimized for both flow cytometry and confocal microscopy. The dye integrates robustly into the OMV membrane, offering stable fluorescence suitable for reliable detection and quantification in flow cytometry and high-resolution imaging in confocal microscopy.

### 4.6. Flow Cytometry Analysis for Escherichia coli OMVs Stained with B2

B2-stained OMVs were analyzed using the small particle detector (SPD) incorporated in the FACSymphony™ A1 flow cytometer (BD Biosciences, Franklin, NJ, USA). Fluorescence signals were detected using a 405 nm excitation laser, with optimal B2 fluorescence recorded in the BV421 channel. All measurements adhered to the manufacturer’s protocol. Control samples included phosphate-buffered saline (PBS) alone and PBS mixed with B2 (without OMVs) to establish the SP-SSC-A threshold alongside a positive control sample of stained OMVs. During acquisition, 50,000–100,000 events were recorded per sample. Labeling efficiency (%) was calculated in FlowJo (v10) as (number of events in the B2 positive gate [Q2])/(total events in the SP-SSC-A gate [Q1 + Q2 + Q3 + Q4]) × 100. Each condition was performed on *n* = 4 independent OMV preparations, and results are reported as mean ± standard error (SE).

### 4.7. Confocal Microscopy Analysis of HT-29 Cells with Escherichia coli OMVs Stained with B2

HT-29 cells (ECCAC 91072201), a human colorectal adenocarcinoma line widely used in biomedical research to investigate intestinal epithelial biology and interactions with outer membrane vesicles (OMVs) [92,93], were seeded onto glass coverslips in a 24-well plate at a density of 2 × 10^5^ cells per well. The cells were then incubated overnight at 37 °C with 5% CO_2_ for adherence and initial growth. Following incubation, cells were washed three times with PBS (phosphate-buffered saline, Gibco, Thermo Fisher, Waltham, MA, USA) to remove the residual medium. Next, epithelial cells were incubated for 3 h at 37 °C with OMVs isolated from *Escherichia coli* strains BL21 or O157 (100 µg/mL according to their protein content), which had been pre-labeled with B2.

Following incubation, the cells were washed thoroughly with PBS to remove unbound OMVs or B2. The cells were then fixed with 4% paraformaldehyde (PFA, Merck, Darmstadt, Germany) for 20 min at room temperature to preserve cellular structures. After fixation, cells were washed four times with PBS (phosphate-buffered saline, Gibco, Thermo Fisher, Waltham, MA, USA). Plasma membrane staining was performed using Alexa Fluor^®^ 647-conjugated wheat germ agglutinin (WGA, Alexa Fluor^®^ 647 conjugate, Thermo Fisher, Waltham, MA, USA) at a concentration of 5 μg/mL for 15 min, which selectively binds to membrane glycoconjugates, enhancing cell membrane visualization.

Finally, the coverslips were mounted on glass slides using Fluoromount-G mounting medium (Thermo Fisher, Waltham, MA, USA) to ensure sample stability and fluorescence preservation. The slides were examined using a confocal microscope, Leica TCS Sp8 DMi8, loaded with two lasers (405 nm for B2 and 638 nm for Alexa Fluor^TM^ 647). Each laser’s PMT was open at 410–550 nm for the B2 channel and 643–799 nm for the WGA channel. Confocal images were acquired and processed using the LASX software version 3.1.5 for subsequent analysis.

### 4.8. Fluorophore Emission Spectra Measurement

The emission spectra for Figure 3 and Figure 6 were measured using a Synergy H1 microplate reader (BioTek, Winooski, VT, USA). For Figure 3B, the OMVs were stained with B2 as described above, the samples were excited at 350 nm, and an emission scan was performed across a range of wavelengths to determine the fluorescence profile of OMVs labeled with B2. For Figure 6, two excitation wavelengths were used: 405 nm for Figure 6A and 638 nm for Figure 6B, measuring an emission scan of Hoechst (3 µg/mL, Thermo Fisher, Waltham, MA, USA), B2 (5 µg/mL, 15.46 µM, [25]), DiO (1% *v*/*v*, Thermo Fisher, Waltham, MA, USA), Alexa Fluor^TM^ 555 (5 µg/mL, Thermo Fisher, Waltham, MA, USA), and Alexa Fluor^TM^ 647 (5 µg/mL, Thermo Fisher, Waltham, MA, USA).

### 4.9. Quantum Chemical Studies

Theoretical computations were performed using density functional theory (DFT) with B3LYP functional hybrid exchange/correlation (XC) functional, which includes the non-local exchange term, with three parameters of Becke and the correlation term of Lee-Yang-Parr [53,94]. The Gaussian basis set def2-TZVPP was used. For the Fukui analysis, the B2 structure was optimized using the Orca 5.0.4 computational package using DFT. Frequency calculation was also performed with the same level of theory to verify that the optimized structure was at a global minimum. To generate the electron density of the different species (anionic, neutral, and cationic), the BP86 functional with the def2-TZVPP basis set and Grimme’s D3 dispersion correction was used [95]. The keyword keepdens was used to keep the electron density. The density of the different species was stored in cube format using the Orca plot tool, and a density subtraction operation was performed in Chemcraft 1.8, according to the following equations:

$$f^{+}\left(r\right)=\rho_{N+1}\left(r\right)-\rho_{N}(r)$$

$$f^{-}\left(r\right)=\rho_{N}\left(r\right)-\rho_{N-1}(r)$$

To reveal possible non-covalent interactions, such as hydrogen bonds, steric repulsion, and van der Waals interactions, a non-covalent interaction index (NCI) was performed. NCI is based on electron density and its derivatives, which enables the identification of non-covalent interactions on the reduced density gradient (S) at low-density regions (ρ). For more details, see [53,96,97].

### 4.10. Molecular Dynamics Simulations of B2 Interacting with a Model OMV Membrane

An asymmetric, heterogeneous lipid bilayer representing the outer membrane of an *E. coli* K12 strain was generated using CHARMM-GUI [98], following the proportions specified previously [99]. This model features a complex assembly, with an outer leaflet composed of lipopolysaccharides (LPS) and an inner leaflet consisting of phospholipids. The LPS layer incorporates critical components (lipid A, core oligosaccharides, and O-antigen), which are essential for maintaining the structural integrity and functionality of the outer membrane. The inner leaflet was made up of 75 molecules of 1-palmitoyl-2-(9-hexadecenoic acid)-phosphatidylethanolamine (PPPE), 20 molecules of 1-palmitoyl-2-(11-octadecenoic acid)-phosphatidylglycerol (PVPG), and 4 molecules of 1′-[1-palmitoyl-2-(11-octadecenoic acid)]-2′-[1-palmitoyl-2-(11-octadecenoic acid)]-cardiolipin (PVCL2). In contrast, the outer leaflet included 34 lipopolysaccharides mimicking an *E. coli* K12 R1 with one O-antigen terminal unit. To evaluate the diffusion potential of the B2 molecules, six units were randomly positioned 20 Å away from the outer membrane. The overall dimensions of the membrane system were 100 × 100 Å, resulting in a comprehensive system containing approximately 135,000 atoms. To ensure accurate and consistent simulations of our membrane system, we followed the CHARMM-GUI protocol [98]. Three independent simulation configurations were generated and subjected to an extended equilibration phase, without restraints, followed by a 1 µs production phase using AMBER24 [100].

## 5. Conclusions

Overall, these observations demonstrate that B2’s dual hydrophobic and polar interactions with OMV membranes endow it with exceptional retention under dynamic conditions, translating into high labeling specificity and minimal background interference. Our emission spectroscopy, flow cytometry, and confocal microscopy data collectively confirm that B2 integrates uniformly into vesicle bilayers, resists aggregation, and generates bright, stable signals across both FACSymphony™ small-particle detection and high-resolution imaging modalities. Such performance underscores B2’s immediate utility for advancing quantitative and spatial studies of bacterial vesicle biology and host–pathogen interactions.

Perhaps more importantly, the benzimidazole core of B2 represents a versatile chemical scaffold for the rational design of next-generation OMV-targeted fluorophores. By strategically modifying this backbone (introducing red- or far-red-shifted chromophores to expand multicolor compatibility or appending hydrophilic moieties to further suppress aggregation), future derivatives can be tailored for enhanced marker stability, tunable spectral properties, and even lower non-specific binding. In this way, B2 provides not only a robust tool in its own right but also a platform upon which a broader suite of optimized probes may be built. We, therefore, anticipate that continued chemical optimization and application-driven refinement of the B2 scaffold will yield a new generation of fluorescent markers, further elevating the fidelity and scope of OMV-based analyses.

## Data Availability

The original contributions presented in this study are included in the article/Supplementary Material. Further inquiries can be directed to the corresponding authors.

## Supplementary Materials

The following supporting information can be downloaded at: https://www.mdpi.com/article/10.3390/ijms26104682/s1.
