# Supplementary material for: Benzimidazole-Derived B2 as a Fluorescent Probe for Bacterial Outer Membrane Vesicle (OMV) Labeling: Integrating DFT, Molecular Dynamics, Flow Cytometry, and Confocal Microscopy
Source: Int J Mol Sci. 2025 May 14;26(10):4682. doi: 10.3390/ijms26104682 (PMC12112470; doi:10.3390/ijms26104682)
Supplement: Supplementary file 1 [file ijms-26-04682-s001.zip › ijms-3598695-supplementary.pdf]

Benzimidazole-Derived B2 as a Fluorescent Probe for Bacterial Outer Membrane Vesicle (OMV) Labeling: Integrating DFT, Molecular Dynamics, Flow Cytometry, and Confocal Microscopy

Francisco Parra [1,2], Alexander Carreño [3,*], Evys Ancede-Gallardo [3], Diana Majluf [1], Jorge A. Soto [4], Romina V. Sepúlveda [5,6], Daniel Aguayo [7], María Carolina Otero [8], Iván L. Calderón [9], Fernando Gil [10,11] and Juan A. Fuentes [1,*]

[1] Laboratorio de Genética y Patogénesis Bacteriana, Centro de Investigación de Resiliencia a Pandemias, Facultad de Ciencias de la Vida, Universidad Andres Bello, Santiago 8370186, Chile; f.parralathrop@uandresbello.edu (F.P.); dmajlufosorio@gmail.com (D.M.)
[2] Doctorado en Biotecnología, Facultad de Ciencias de la Vida, Universidad Andres Bello, Santiago 8370186, Chile
[3] Laboratory of Organometallic Synthesis, Departamento de Ciencias Químicas, Facultad de Ciencias Exactas, Universidad Andres Bello, Santiago 8370186, Chile; eancedeg@gmail.com
[4] Millennium Institute on Immunology and Immunotherapy, Laboratorio de Inmunología Traslacional, Centro de Investigación de Resiliencia a Pandemias, Facultad de Ciencias de la Vida, Universidad Andres Bello, Santiago 8370186, Chile; jorge.soto.r@unab.cl
[5] Center for Bioinformatics and Integrative Biology (CBIB), Facultad de Ciencias de la Vida, Universidad Andres Bello, Santiago 8370146, Chile; romina.sepulveda@unab.cl
[6] ANID—Millennium Nucleus in Data Science for Plant Resilience (PhytoLearning), Facultad de Ciencias de la Vida, Universidad Andres Bello, Santiago 8370146, Chile
[7] Instituto de Tecnología para la Innovación en Salud y Bienestar (ITISB), Facultad de Ingeniería, Universidad Andres Bello, Viña del Mar 2531015, Chile; daniel.aguayo@unab.cl
[8] Escuela de Química y Farmacia, Facultad de Medicina, Universidad Andres Bello, Santiago 7591538, Chile; maria.otero@unab.cl
[9] Laboratorio de RNAs Bacterianos, Centro de Investigación de Resiliencia a Pandemias, Facultad de Ciencias de la Vida, Universidad Andres Bello, Santiago 8370186, Chile; lcalderon@unab.cl
[10] School of Medicine, Faculty of Medicine, Universidad de los Andes, Santiago 7620001, Chile; frgil@uandes.cl
[11] Microbiota-Host Interactions & Clostridia Research Group, Center for Biomedical Research and Innovation (CIIB), Universidad de los Andes, Santiago 7620001, Chile
* Correspondence: alexander.carreno@unab.cl (A.C.); jfuentes@unab.cl (J.A.F.)

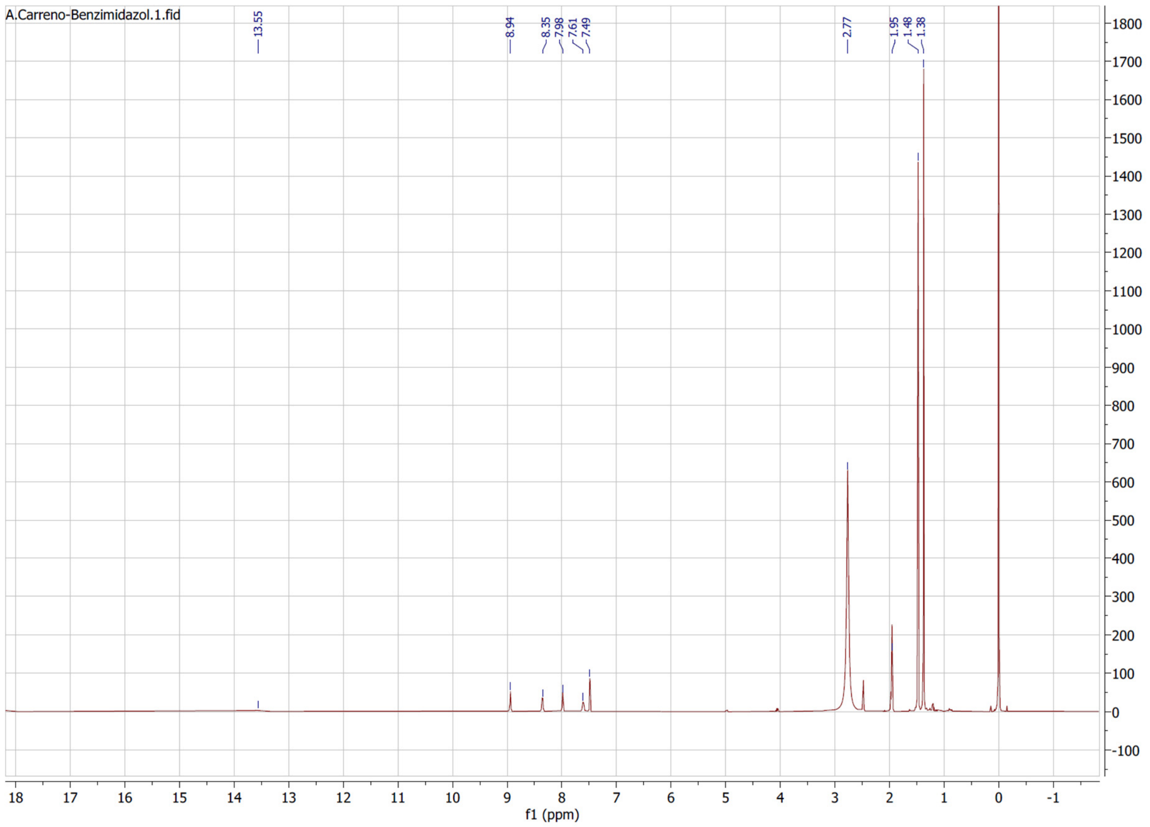

Figure S1. ^{1}H NMR of B2 at 400 MHz and 25 °C, with samples dissolved in deuterated DMSO.

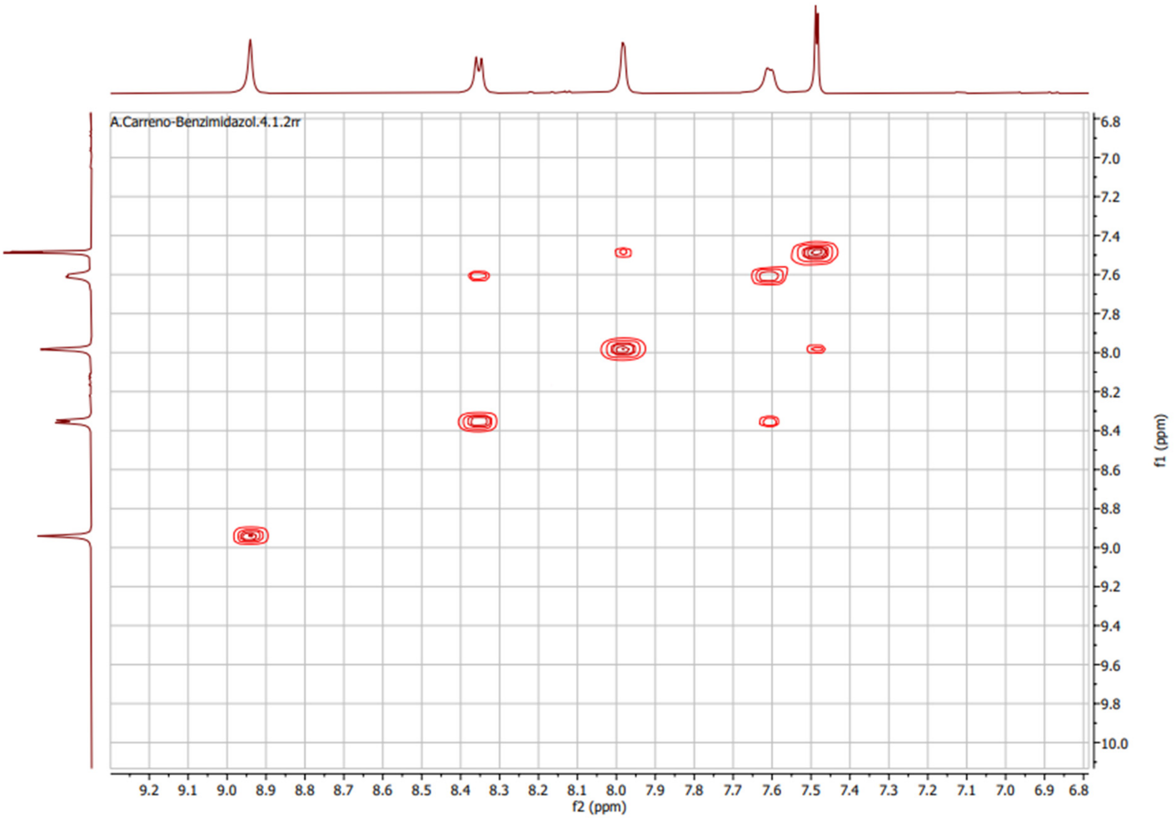

Figure S2. HHCOSY of B2 at 400 MHz and 25 °C, with samples dissolved in deuterated DMSO.

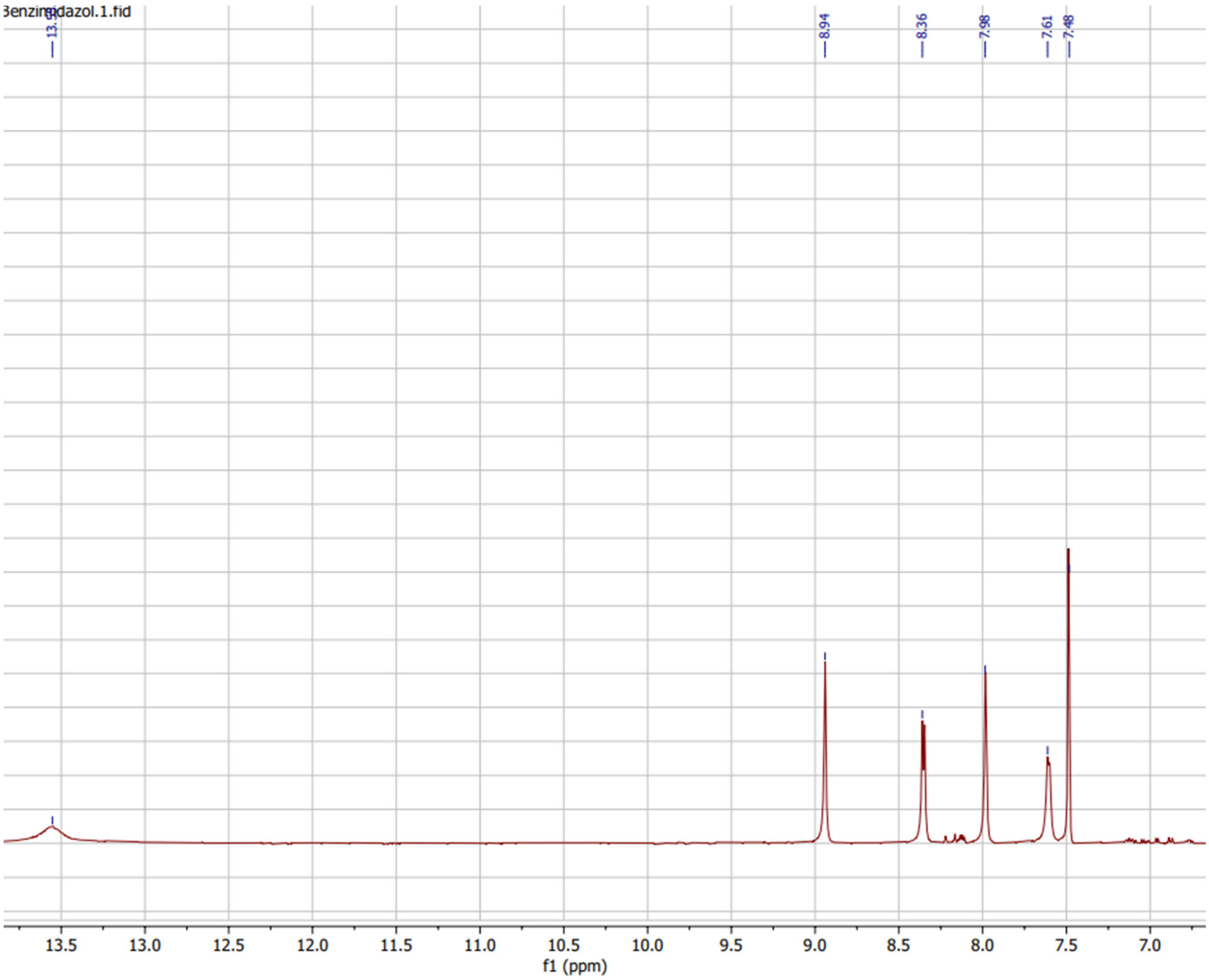

Figure S3. Expanded aromatic zone of ^{1}H NMR spectrum of B2 at 400 MHz and 25 °C, with samples dissolved in deuterated DMSO.

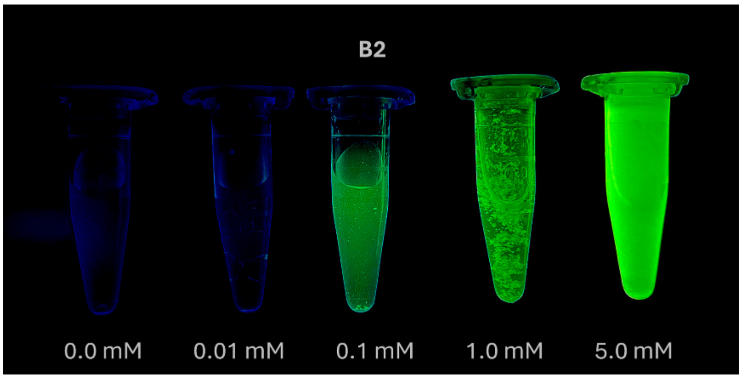

Figure S4. UV-transillumination images illustrating aggregation and fluorescence of B2 diluted in PBS in the absence of OMVs. As shown in the figure, the fluorescence is dependent on the B2 concentration. At 0.1 mM, B2 yields a homogeneous green fluorescence with no visible particulate formation, confirming full solubilization at the concentration used for OMV labeling. In contrast, fluorescent aggregates become evident at concentrations ≥ 1.0 mM, indicating that precipitates in PBS become detectable only at higher dye concentrations.

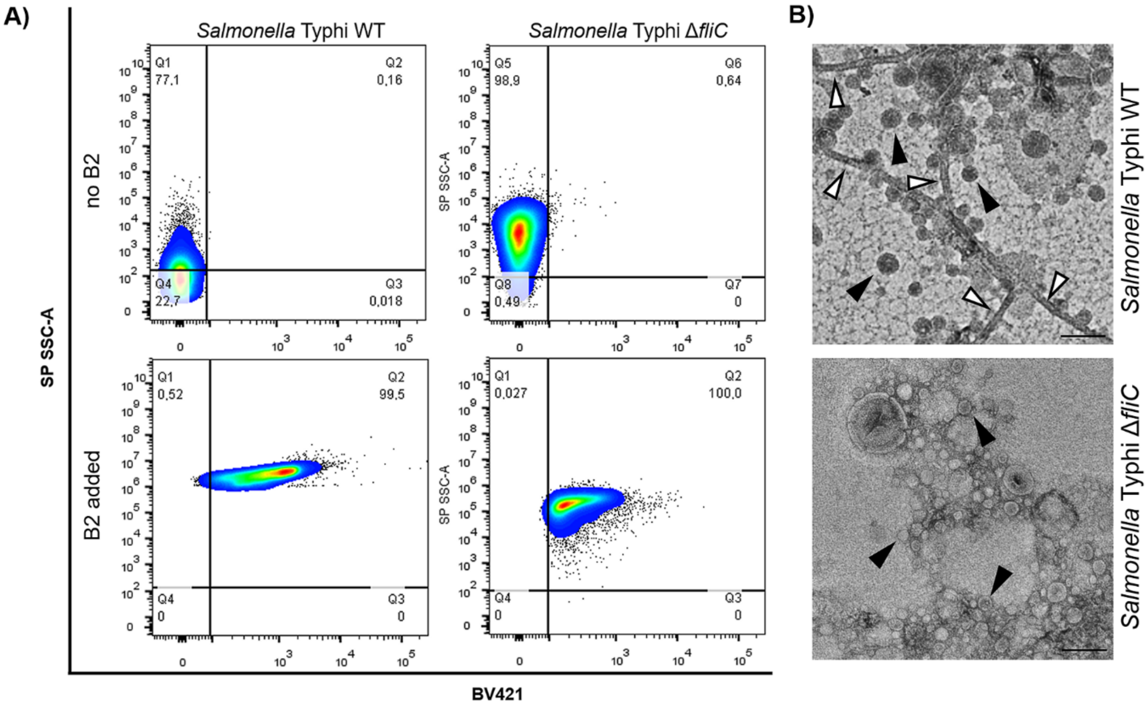

Figure S5. A) Flow cytometry analysis of OMVs labeled with B2. Flow cytometry was performed to analyze OMVs extracted from *Salmonella enterica* subsp. *enterica* sv. Typhi strain STH2370 WT and Δ*fliC* (a strain lacking flagella) after labeling with B2. (Top row) Controls showing OMVs with no B2. (Bottom row) B2-labeled OMVs show almost 100% fluorescence,independently of the presence of flagella. Analyses were performed using a FACSymphony™ A1 flow cytometer (BV421 channel) and FlowJo software v10. B) Transmission electron microscopy (TEM) of OMVs produced by *S.* Typhi WT and Δ*fliC*. Black arrowheads: OMVs. White arrowheads: flagella. Bacteria were cultured in LB to OD_{600} = 1.1 before extracting OMVs. The bar corresponds to 100 nm. A representative experiment is shown (n = 3).
